# Supplementary material for: Optimization of 4,6-α and 4,3-α-Glucanotransferase Production in Lactococcus lactis and Determination of Their Effects on Some Quality Characteristics of Bakery Products
Source: Foods. 2024 Jan 29;13(3):432. doi: 10.3390/foods13030432 (PMC10855804; doi:10.3390/foods13030432)
Supplement: Supplementary file 1 [file foods-13-00432-s001.zip › foods-2835192-supplementary.pdf]

Table S1. Ingredients used in the production of bread and bun

Ingredients	Bread (%)	Bun (%)
**Flour**	100	100
**Water**	x-2	40
**Salt**	1	2
**Yeast**	2	5
**Fat**	-	50
**Sugar**	-	5
**Egg**	-	20

X: Amount of water determined in the farinograph
